# *Ruppia mongolica* (Ruppiaceae), a new species from Inner Mongolia (China), based on morphological and genetic data

**DOI:** 10.1002/ece3.9989

**Published:** 2023-04-07

**Authors:** Yang Zou, Xiaofan Wang, Xinwei Xu, Dan Yu

**Affiliations:** ^1^ National Field Station of Freshwater Ecosystem of Liangzi Lake, College of Life Sciences Wuhan University Wuhan China; ^2^ Department of Ecology, College of Life Sciences Wuhan University Wuhan China

**Keywords:** Inner Mongolia, karyotype analysis, new species, phylogenetic analysis, *Ruppia*

## Abstract

*Ruppia mongolica* Y. Zou & X.W. Xu, a new species from Inner Mongolia, China, is described and illustrated. The phylogenetic position of the new species within the genus was analyzed based on eight chloroplast DNA fragments and an ingroup sampling of all Eurasian species of *Ruppia*. The results showed that *R. mongolica* formed a separate branch between *R. sinensis* and the clade of *R. maritima*, *R. brevipedunculata*, *R. drepanensis*, and *R. cirrhosa*. Based on molecular and geographical evidence, our study reveals that *R. mongolica* is closely related to *R. sinensis* and *R. brevipedunculata* but differs from the former in the length and shape of the peduncle and seed size, and from the latter in the length of the peduncle, number of carpels per flower, and seed size. In addition, the karyotype analysis revealed that *R. mongolica* is octoploid, which is first reported within *Ruppia*, further supporting *R. mongolica* as a new species.

## Graphical abstract

This study reported a new species of *Ruppia* discovered in two localities in Inner Mongolia, China. We identified it as a new species based on morphological comparison, phylogenetic analysis and karyotype analysis.

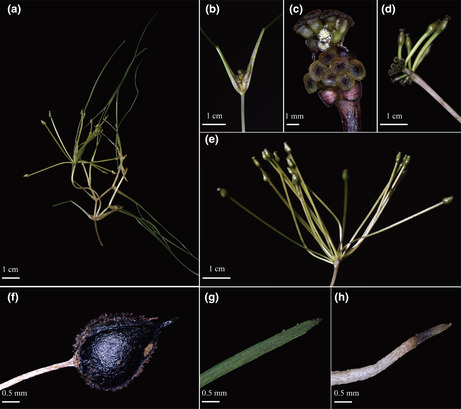

## 1 INTRODUCTION

Ruppiaceae is a family in the order Alismatales and is closely related to two seagrass families Cymodoceaceae and Posidoniaceae (Angiosperm Phylogeny Group IV, 2016). *Ruppia* L. is the only genus in Ruppiaceae that exhibits a worldwide distribution and inhabits coastal lagoons, saltmarsh ponds, inland brackish, and saline waters (Calado & Duarte, 2000; Menéndez et al., 2002; Short et al., 2007). This genus is an important primary producer and has a high potential for biomonitor and phytoremediation of heavy metals in these waters (Gu et al., 2021; Zhao & Wu, 2008). The number of species within *Ruppia* has always been controversial (Cook, 1990), and five accepted species, *Ruppia maritima* L., *Ruppia cirrhosa* (Petagna) Grande, *Ruppia megacarpa* Mason, *Ruppia polycarpa* Mason, and *Ruppia tuberosa* Davis & Tomlinson, were listed in the review on the taxonomy of *Ruppia* based on morphological traits (Zhao & Wu, 2008).

In recent years, several new species have been reported based on morphological and/or genetic data, e.g., *Ruppia sinensis* Shuo Yu & den Hartog and *R. brevipedunculata* Shuo Yu & den Hartog from China (Yu & den Hartog, 2014), *R. bicarpa* Y. Ito et Muasya from South Africa (Ito et al., 2015), *R. mexicana* den Hartog and van Tussenbroek from Mexico (Den Hartog et al., 2016), suggesting that the diversity of *Ruppia* has been underestimated. There are a total of 11 accepted species within *Ruppia* in the world (POWO, 2022). Among these species, two are worldwide, *R. maritima* and *R. cirrhosa*, while other species are limited to one country or region (POWO, 2022).

Recently, during field investigations in Inner Mongolia, China, we collected distinct specimens of *Ruppia* from Urad Front Banner (41.089° N, 108.929° E) and Tumote Right Banner (40.495° N, 110.785° E). Studies based on specimens and literature examination comparing these specimens and two Chinese coastal species, *R. sinensis* and *R. brevipedunculata*, as well as molecular phylogenetic analysis and karyotype analysis, revealed it to be an undescribed species. We herein describe, discussed, and illustrate the new species.

## 2 MATERIALS AND METHODS

### 2.1 Morphological analysis

Specimens of the new species were collected from Urad Front Banner, Inner Mongolia, China. Some living plants were introduced and cultivated at the College of Life Sciences, Wuhan University. Roots, leaves, flowers, and seeds from living plants were examined using a stereoscopic microscope (Olympus U‐TV1XC, Tokyo, Japan). The morphological data of two Chinese coastal species, *R. sinensis* and *R. brevipedunculata*, were obtained from the published paper (Yu & den Hartog, 2014).

### 2.2 Karyotype analysis

Mature seeds were collected from the natural population in Urad Front Banner, Inner Mongolia, China. They were incubated on wet filter paper (10 ppt) in Petri dishes at 25/20°C (12/12 h) and with a 12 h daily light period for 3 days, during which time germination occurred. Root tips were pretreated with an ice‐water mixture in a dark room for 24 h. After incubation, the tips were fixed in Carnoy I solution (3:1 ethanol: glacial acetic acid) at 4°C for at least 4 h. They were then digested at 37°C in a combination (1:1) of 2% cellulase and 2% pectinase for 30–60 min before staining with an improved carbol fuchsin solution and squashed for cytological observation (Carr & Walker, 1961). Standard liquid nitrogen method was used to make permanent slides that were preserved at Wuhan University. The photo micrographs were taken using an Axio Imager A1 microscope (Zeiss, Germany). We also collected seeds of *R. sinensis* from Jinzhou, Liaoning, China, and carried out the same karyotype analysis.

### 2.3 Molecular analysis

The fresh and healthy young leaves of five individuals of the new species (one from Urad Front Banner and four from Tumote Right Banner) were used for genomic DNA extraction. Paired‐end Illumina libraries with insert sizes of ~300 bp were constructed from the extracted DNA using the NEBNext Ultra DNA Library Prep Kit according to the manufacturer's instructions and sequenced on the NovaSeq 6000 platform. Fastp v.0.20.0 was used for quality control and adapter trimming to obtain high‐quality reads (Chen et al., 2018). A total of eight chloroplast DNA fragments, six intergenic spacer regions (*atpA‐atpF*, *psbC‐trnS*, *infA‐rps8*, *psbJ‐psbL*, *rpl36‐rps11*, and *rpl14‐rps8*) and two microsatellites (ccmp2 and ccmp10) from Triest and Sierens (2014) (Table S1), were used for phylogenetic analyses. Sequences of five haplotypes/lineages (A‐E) in three Eurasian species, *R. maritima*, *R. cirrhosa*, and *R. drepanensis* (Triest & Sierens, 2014) were downloaded from GenBank. Sequences of five individuals of the new species were obtained in two steps: Firstly, the chloroplast genome of each individual was obtained from sequence reads using GetOrganelle v.1.6.3a (https://github.com/Kinggerm/GetOrganelle) with the chloroplast genome of *R. sinensis* (MN233650, Yu et al., 2019) as the reference, and annotated using CPGAVAS2 (Shi et al., 2019); Secondly, the chloroplast genome was aligned against sequences of the five haplotypes using Geneious v.20.2.2 (https://www.geneious.com/). Sequences of two Chinese *Ruppia* species and two outgroups were obtained from their chloroplast genomes, *R. sinensis* (MN233650, Yu et al., 2019), *R. brevipedunculata* (MN736637, Yu et al., 2020), *Zostera marina* (MF370229, Xing & Guo, 2018), and *Potamogeton perfoliatus* (NC_029814, Luo et al., 2016). Sequences were aligned using MAFFT v.7.308 (Katoh & Standley, 2013). Sequences of the eight chloroplast DNA fragments were concatenated using PhyloSuite v.1.1.15 (Zhang et al., 2020). Gaps were treated as missing data. The most suitable model for the combined data was determined using ModelFinder implemented in PhyloSuite. The maximum likelihood (ML) trees were constructed using IQ‐TREE implemented in PhyloSuite. The support of the branches was estimated using 1000 bootstrap replicates. Phylogenetic tree was visualized in iTOL v.6.6 (https://itol.embl.de).

## 3 RESULTS

### 3.1 Morphological characters and chromosome number

**FIGURE 1:**
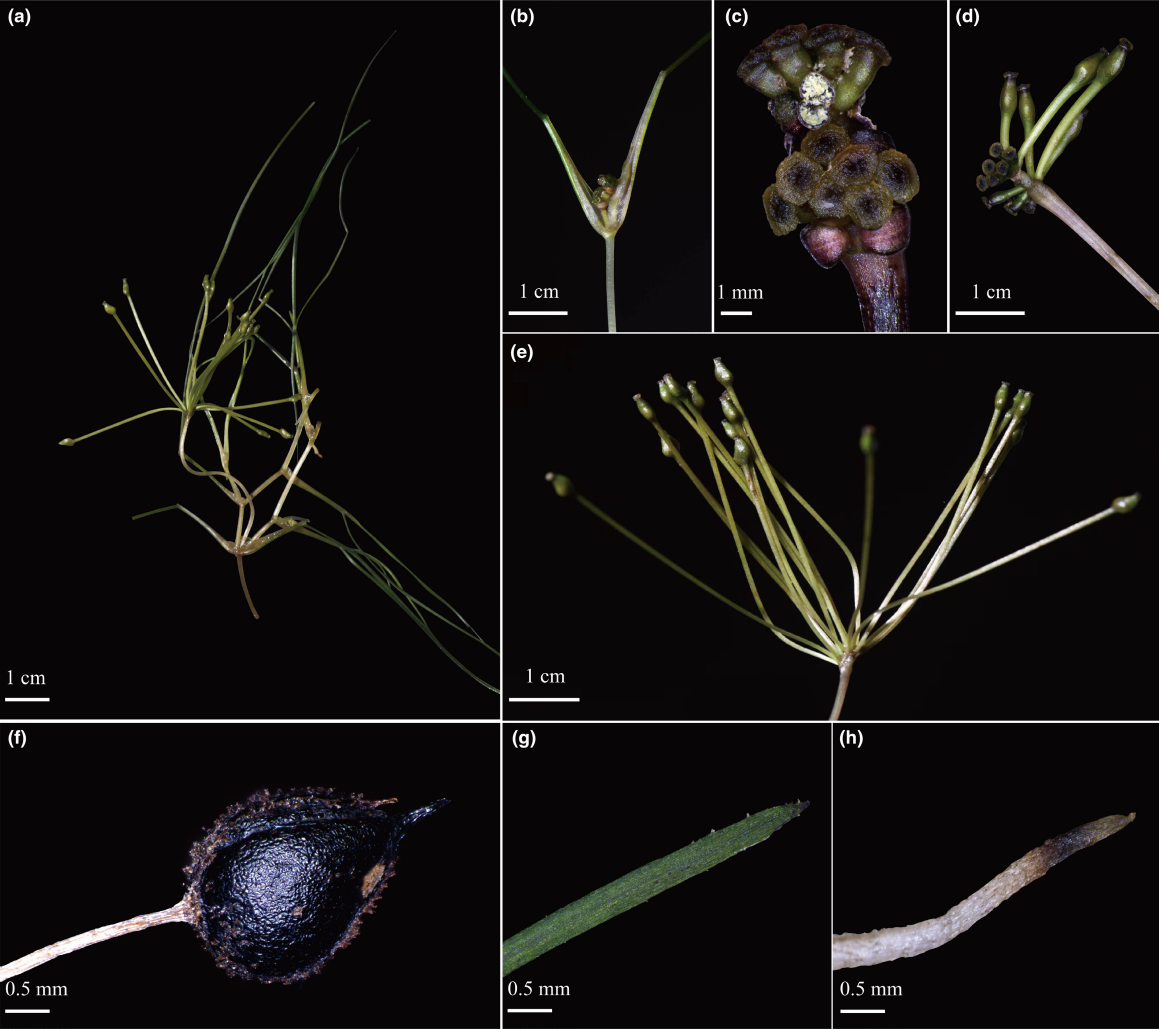
*Ruppia mongolica* Y. Zou & X.W. Xu, sp. *nov*. (a) Habit; (b) leaf sheath; (c) inflorescence; (d) immature fruits; (e) ripe fruits; (f) seed; (g) leaf apex; (h) root apex.

**FIGURE 2:**
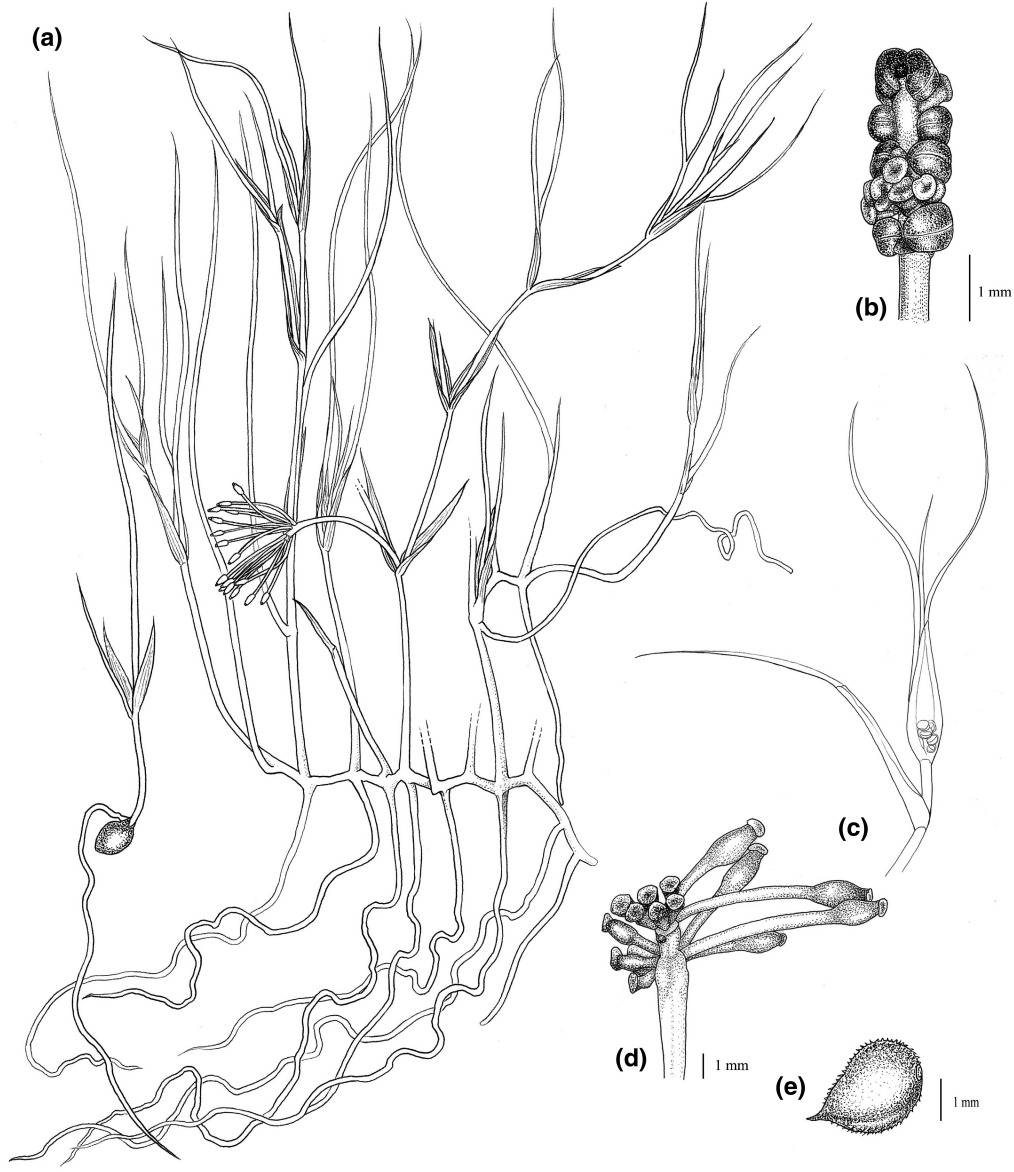
Illustration of *Ruppia mongolica* Y. Zou & X.W. Xu, sp. *nov*. Drawn by Ya Li. (a) Plant; (b) inflorescence; (c) a branch showing a complete leaf at the bottom and an inflorescence in a leaf sheath; (d) immature fruits; (e) seed.

**FIGURE 3:**
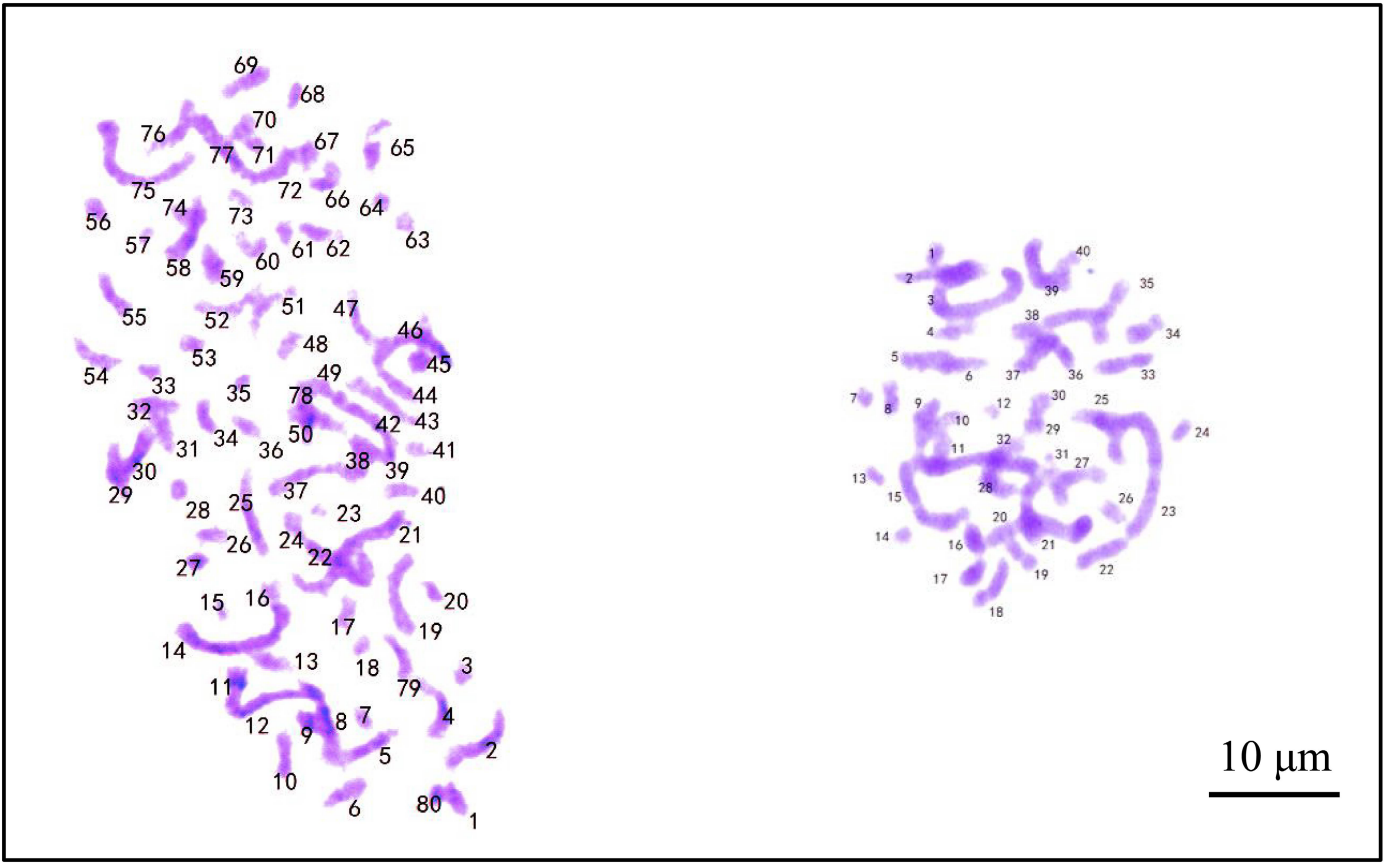
Metaphase chromosomes of *Ruppia mongolica* Y. Zou & X.W. Xu, sp. *nov* (left) and *Ruppia sinensis* (right).

The morphological characters of the new species differ from all species previously described in *Ruppia*. The short peduncle of the new species (1.8–3.8 cm, Figures 1 and 2) makes it quite distinct from other *Ruppia* species, except for *R. maritima* and *R. brevipedunculata,* which have similar peduncle length. However, the number of carpels of the new species (6–8, Figures 1 and 2) is evidently more than the latter two species (2–5 in *R. maritima* and 4 in *R. brevipedunculata*). The new species can be distinguished from two Chinese species, *R. sinensis* and *R. brevipedunculata*, in several traits: differing from *R. sinensis* in the length and shape of peduncle and seed size, and from *R. brevipedunculata* in the length of peduncle, number of carpels per flower and seed sizes (Table 1). The karyotype analysis revealed that the chromosome number of *R*. *mongolica* is 2n = 8x = 80, while that of *R. sinensis* is 2n = 4x = 40 (Figure 3).

**TABLE 1:** Main distinctive morphological characters comparing *Ruppia mongolica* with related species.

Characters	*R. mongolica*	*R. sinensis*	*R. brevipedunculata*
Shape of the peduncle	straight	spirally coiled	straight
Length of the peduncle	1.8–3.8 cm	1.4–10.8 cm	0.1–0.7 cm
Number of Carpels	6–8	4–10	4
Length of the Podetium	2.4–3.5 cm	0.5–3.5 cm	0.4–2.5 cm
Length of the seed	2.2–3.1 mm	1.5–2.5 mm	2.1–2.6 mm
Width of the seed	1.7–2.4 mm	1.4–2.1 mm	1.1–1.6 mm

### 3.2 Phylogenetic analysis

We obtained complete chloroplast genomes of four individuals of *R. mongolica* from Tumote Right Banner and submitted one of them to GenBank because they are identical. Although the chloroplast genome of the individual from Urad Front Banner was incomplete, its sequences of the eight chloroplast DNA fragments were identical as those of individuals from Tumote Right Banner. Therefore, only one operational taxonomic unit of the new species was included in the phylogenetic analysis.

**FIGURE 4:**
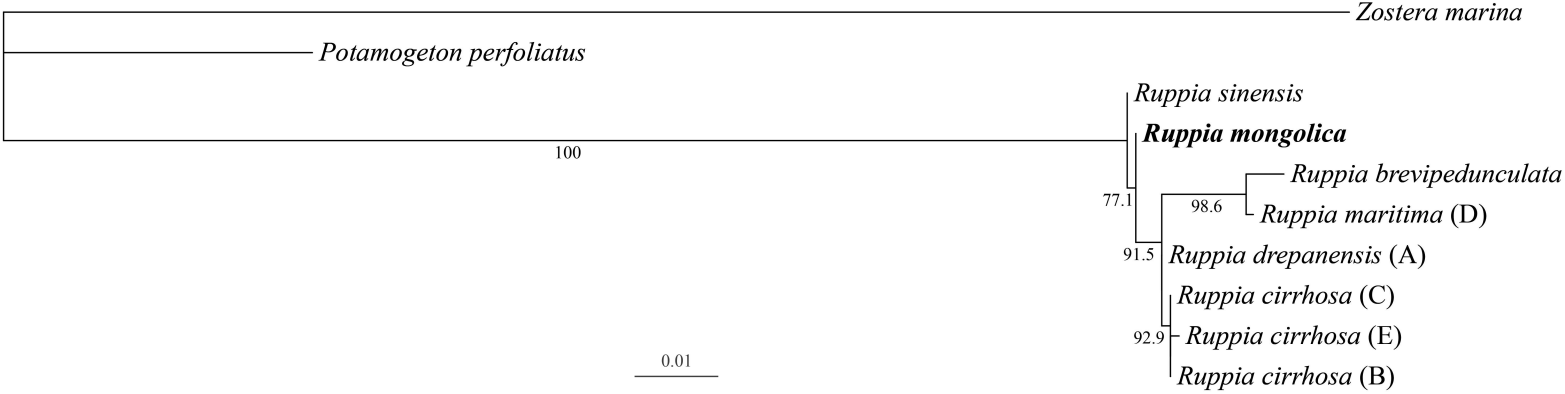
Phylogram showing the best tree of maximum likelihood analysis of *Ruppia*. Values on the branches are maximum likelihood bootstrap.

The length of the aligned sequences of the eight chloroplast DNA fragments was 1240 bp. The best‐fit model of the concatenated matrix was GTRCAT. The ML analysis found the best tree with a log‐likelihood value of −2891.998. *Ruppia* was found monophyletic (Bootstrap value (BS) = 100), and the first divergent branch was *R. sinensis* followed by a clade (BS = 77.1) with *R. mongolica* and a lineage (BS = 91.5) containing *R. martinima*, *R. brevipedunculata*, *R. drepanensis*, and *R. cirrhosa* (Figure 4), which verifies that *R. mongolica* is genetically distinct and supports its status as a new species.

### 3.3 Taxonomic treatment

*Ruppia mongolica* Y. Zou & X.W. Xu, *sp. nov* (Figures 1 and 2).

Type: CHINA. Inner Mongolia: Urad Front Banner, Dashetai Town, in brackish waters, 41°5′20″ N, 108°55′44″ E, alt. 1007 m, 11 Sept. 2022, *X.W. Xu & Y. Zou 2,022,091,101* (holotype: WHU; isotype: WHU).

#### 3.3.1 Diagnosis

The new species is most closely related to *R. sinensis* and *R. brevipedunculata* but differs from the former in its short and straight peduncles (vs. long and spirally coiled) and 2.2–3.1‐mm‐long seeds (vs. 1.5–2.5‐mm‐long), and differs from the latter in 1.8–3.8‐mm‐long peduncles (vs. 0.1–0.7‐mm‐long), 6–8 carpels per flower (vs. 4 carpels), and 1.7–2.4‐mm‐wide seeds (vs. 1.1–1.6‐mm‐wide).

#### 3.3.2 Description

Aquatic plant. Annual, 30–50‐cm‐long, depending on the water depth, slender, always branched. Rhizome creeping, internodes 1–8‐cm‐long, 0.8–1.8‐mm‐thick, monopodial; at each node one root grows downward, 1–6‐cm‐long, white with a brown or black tip; length of shoot internodes variable and the lowest shoot internode short and thick. Leaf‐sheath amplexicaulous, 0.8–2‐cm‐long, about 5‐mm‐wide at the base, with two auriculas on the top of the sheath; the sheath is inflated and translucent with a membranous flap; leaves linear, tip acute, up to 21‐cm‐long and 0.3–0.8‐mm‐wide, with a clear midrib; leaf margin near the acute tip with some minute spiny teeth. Inflorescence 2‐flowered with a single axis surrounded by leaf sheath, initially subsessile; the two flowers are inserted opposite, each flower bisexual, perianth absent. Androecium with four stamens, each stamen with one bilocular anther. Gynoecium with 6–8 carpels, free, bottle‐like ovary with a fleshy peltate stigma. After fertilization, peduncles elongate and appear from the leaf sheath, usually straight, 1.8–3.8‐cm‐long. Fruit asymmetric, ovate, brown with a podogyne; podetium 2.4–3.5‐cm‐long, easily shed from ovule portion of seed; endocarp hard and black after exocarp decayed. Seed with a beak about 0.3–0.7 mm; 2.2–3.1‐mm‐long (including the beak length), 1.7–2.4‐mm‐wide. Chromosome number 2n = 8x = 80.

#### 3.3.3 Phenology

Flowering: August–September, Fruiting: September–October.

#### 3.3.4 Distribution and habitat

**FIGURE 5:**
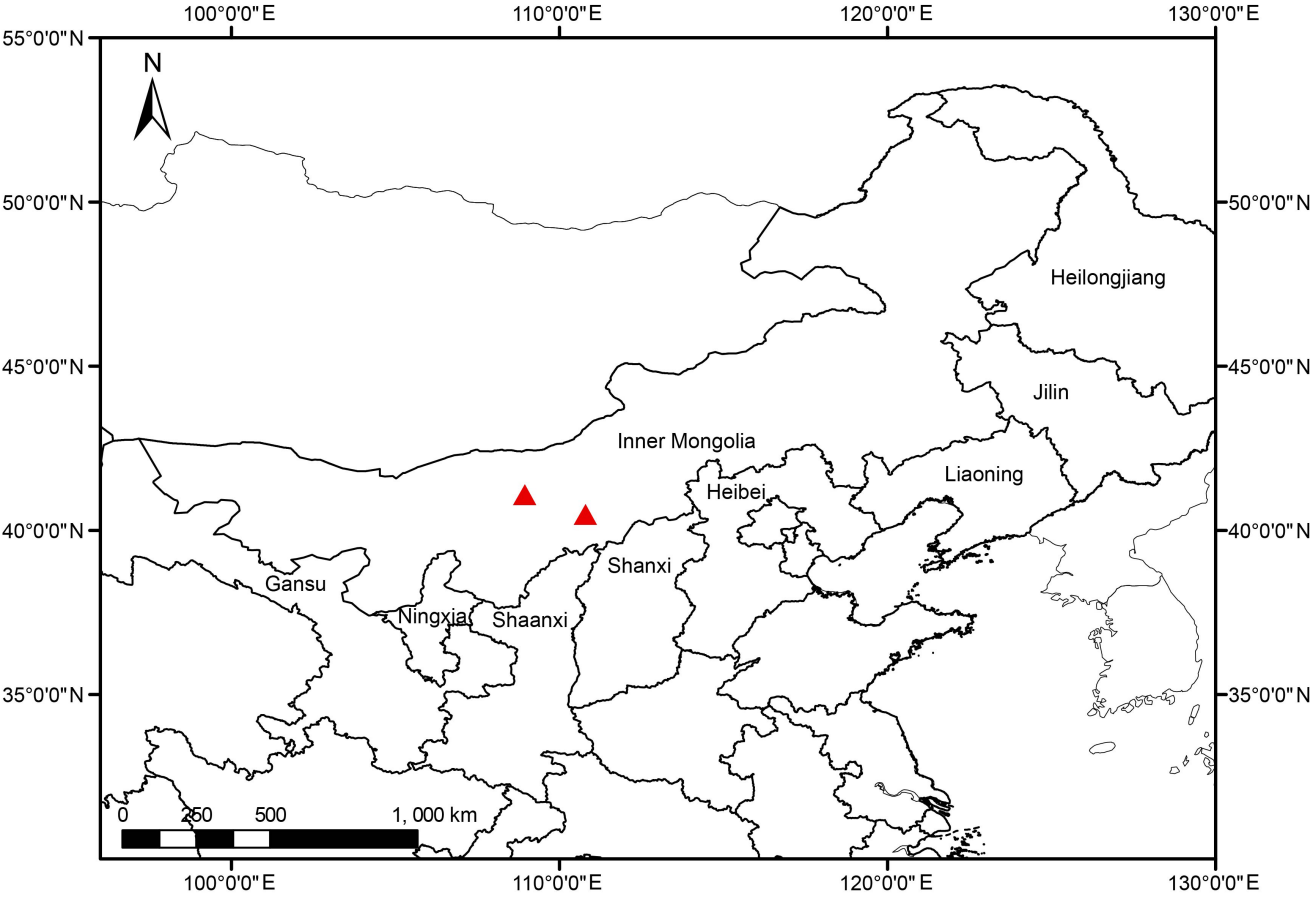
Distribution of *Ruppia mongolica* Y. Zou & X.W. Xu, sp. *nov*.

To date, *Ruppia mongolica* has only been found in the two localities in central Inner Mongolia, China (Figure 5). It grows in shallow stagnant waters with salinity 3–15 ppt.

## 4 DISCUSSION

The characteristics in the reproductive architecture are considered important evidence for delimiting species within the genus *Ruppia* (Hammer & Heseltine, 1988; Jagels & Barnabas, 1989; Kaul, 1993). The new species differ from all species previously described in *Ruppia* by the peduncle morphology, number of carpels per flower, and/or seed size. Among six well‐accepted species, *R. maritima*, *R. drepanensis*, *R. cirrhosa*, *R. polycarpa*, *R. megacarpa*, and *R. tuberosa*, only *R. maritima* has short and not spirally coiling peduncle (<5 cm) (Mannino et al., 2015), which is similar to that of *R. mongolica* (1.8–3.8 cm). However, the number of carpels of *R. mongolica* (6–8) is more than that of *R. maritima* (2–5). The recently described species *R. bicarpa* from South Africa has flowers with only two carpels (Ito et al., 2015), and *R. mexicana* from Mexico (Den Hartog et al., 2016) has spirally coiled long peduncle (5–25 cm) and 2–4 carpels per flower, which are different from those of *R. mongolica*.

Differences in chromosome numbers have been observed in *Ruppia*. Diploid (2n = 20), triploid (2n = 30), tetraploid (2n = 40), and hexaploid (2n = 60) have been reported (Ito et al., 2010; Talavera et al., 1993). Among four newly published species, both *R. mexicana* (Den Hartog et al., 2016) and *R. sinensis* (Figure 3, our data) are tetraploid. Therefore, the octoploid *R. mongolica* is first reported within *Ruppia*.

Although the Australian species *R. polycarpa*, *R. megacarpa*, and *R. tuberosa* and the African species *R. bicarpa* were not included in our phylogenetic analysis, their phylogenetic position was basal and genetically differed from Eurasian species (Ito et al., 2015). Within Eurasian species, *R. mongolica* was reconstructed as a branch between *R. sinensis* and the clade of *R. maritima*, *R. brevipedunculata*, *R. drepanensis*, and *R. cirrhosa* with good support (Figure 4). Therefore, based on molecular and geographical evidence, *R. mongolica* is most closely related to two Chinese species *R. sinensis* and *R. brevipedunculata*. However, distinct characteristics in reproductive architecture between *R. mongolica* and these two species (Table 1) corroborate the recognition of a new species.

*Ruppia* is distributed in coastal areas and inland provinces of China and only one species, *R. maritima*, was recorded in the Flora of China (Guo et al., 2010). By contrast, recent studies based on molecular and morphological data supported populations in coastal areas as two new species, *R. sinensis* and *R. brevipedunculata* (Yu & den Hartog, 2014). Now our study indicates *Ruppia* from two sites in Inner Mongolia as the third new species in China. We also have collected *Ruppia* specimens from other sites in Inner Mongolia and other inland provinces, such as Ningxia, Gansu, and Xinjiang, but we identified these samples as *R. sinensis*, indicating the limited distribution of *R. mongolica*.

## AUTHOR CONTRIBUTIONS

**Yang Zou:** Conceptualization (lead); investigation (lead); software (lead); visualization (lead); writing – original draft (lead). **Xiao‐Fan Wang:** Conceptualization (supporting). **Xinwei Xu:** Conceptualization (supporting); investigation (supporting); writing – review and editing (supporting). **Dan Yu:** Conceptualization (supporting); funding acquisition (lead); methodology (supporting); validation (supporting); writing – review and editing (supporting).

## CONFLICT OF INTEREST STATEMENT

We declare that the named authors have no conflicts of interest, financial or otherwise.

## Supporting information


Table S1.

## Data Availability

All raw sequencing data obtained from this study have been deposited in the Genome Sequence Archive in National Genomics Data Center under the accession number: CRA009765. The chloroplast DNA sequences analyzed for this study can be found in the GenBank with accession number OP723916.
