# Supplementary material for: Ruppia mongolica (Ruppiaceae), a new species from Inner Mongolia (China), based on morphological and genetic data
Source: Ecol Evol. 2023 Apr 7;13(4):e9989. doi: 10.1002/ece3.9989 (PMC10082167; doi:10.1002/ece3.9989)
Supplement: Supplementary file 1 — Table S1. [file ECE3-13-e9989-s001.docx]

**Table S1** Accession numbers of all sequences used in this study.

| Taxa | Chloroplast DNA fragment or chloroplast genome | | | | | | | |
| --- | --- | --- | --- | --- | --- | --- | --- | --- |
|  | *atpA-atpF* | *psbC-trnS* | *infA-rps8* | *psbJ-psbL* | *rpl36-rps11* | *rpl14-rps8* | ccmp2 | ccmp10 |
| *Ruppia drepanensis* (A) | KJ010058 | KJ010062 | KJ010064 | KJ010068 | KJ010071 | KJ010073 | JN113249 | JN113264 |
| *Ruppia cirrhosa* (B) | KJ010058 | KJ010062 | KJ010064 | KJ010067 | KJ010071 | KJ010073 | JN113250 | JN113260 |
| *Ruppia cirrhosa* (C) | KJ010058 | KJ010062 | KJ010064 | KJ010067 | KJ010071 | KJ010073 | JN113252 | JN113263 |
| *Ruppia cirrhosa* (E) | KJ010058 | KJ010062 | KJ010064 | KJ010067 | KJ010071 | KJ010073 | JN113254 | JN113262 |
| *Ruppia maritima* (D) | KJ010060 | KJ010062 | KJ010065 | KJ010069 | KJ010072 | KJ010074 | JN113255 | JN113261 |
|  | Chloroplast genome | | | | Source | | | |
| *Ruppia mongolica* | OP723916 | | | | This study | | | |
| *Ruppia brevipedunculata* | MN736637 | | | | Yu et al., 2020 | | | |
| *Ruppia sinensis* | MN233650 | | | | Yu et al., 2019 | | | |
| *Zostera marina* | MF370229 | | | | Xing and Guo, 2018 | | | |
| *Potamogeton perfoliatus* | NC_029814 | | | | Luo et al., 2016 | | | |

*Some species show no polymorphism in sequences of some chloroplast DNA fragments and have the same accession numbers.
